# Ondansetron and simvastatin added to treatment as usual in patients with schizophrenia: study protocol for a randomized controlled trial

**DOI:** 10.1186/1745-6215-14-101

**Published:** 2013-04-17

**Authors:** Imran B Chaudhry, Nusrat Husain, Mohammad O Husain, Jamie Hallak, Richard Drake, Ajmal Kazmi, Raza ur Rahman, Mohammad M Hamirani, Tayyaba Kiran, Nasir Mehmood, John Stirling, Graham Dunn, Bill Deakin

**Affiliations:** 1 University of Manchester and Lancashire Care Early Intervention Service, The Mount Whalley Road, Accrington BB5 5DE, UK; 2 St Georges University, Cranmer Terrace, London SW 17 ORE, UK; 3 University of San Paulo, Rua da praca Relogio, 109 Sao Paulo, BR 05508-900, Brazil; 4 KPT and Karwan e Hyat “Psychiatric Care and Rehabilitation Cent34 (PCRC)” Near KPT hospital, Keamari, Karachi, Pakistan; 5 Dow University of Health Sciences, Baba-E-Urdu Road, Karachi 74200, Pakistan; 6 Abbassi Shaheed Hospital, Block M, North Nazimabad, Karachi, Pakistan; 7 Pakistan Institute of Learning & Living, D-9, Block I, Northnazimabad, Karachi 74700, Pakistan; 8 Manchester Metropolitan University, All Saints Building, All Saints, Manchester M15 6BH, UK

**Keywords:** Anti-inflammatory, Schizophrenia, Pakistan, Psychosis

## Abstract

**Background:**

Negative symptoms and cognitive deficits are two partially-related features of schizophrenia which have a major negative impact on social function and objective quality of life. Standard drug treatments have little impact on either. There is some evidence that anti-inflammatory treatment may have beneficial effects in schizophrenia and major depression. Statins are cholesterol-lowering agents that have been found to be anti-inflammatory agents and are also known to decrease C-reactive protein (CRP). Ondansetron is a serotonin (5-HT3) receptor antagonist widely used to prevent nausea and vomiting in patients receiving chemotherapy for cancer. Small studies have suggested that ondansetron is effective as an adjunct drug in improving the symptoms of schizophrenia.

**Methods/design:**

This is a two center, six-month, double-blind placebo controlled, factorial design study of ondansetron and/or simvastatin added to treatment as usual for patients suffering from schizophrenia, schizoaffective disorder, psychosis not otherwise specified or schizophreniform disorder. This will be a 2 × 2 design, with 54 patients in each cell, giving a total of 216 patients over three years. There will be a screening, a randomization and seven follow-up visits. Full clinical and neurocognitive assessments will be carried out at baseline (randomization), 14 weeks and at 26 weeks, while the positive and negative syndrome scale (PANSS), pill count and side effects checklist will be carried out at every visit. Simvastatin will be started at 20 mg once daily (OD), this will be increased to 40 mg after four weeks. Ondansetron will be administered in an 8 mg dose.

**Discussion:**

Anti-inflammatory treatments have been shown to have some beneficial effects in schizophrenia. Both simvastatin and ondansetron provide some evidence of a reduction in symptoms compared to treatment as usual. The aim of this study is to establish the degree of improvement in negative symptoms with the addition of ondansetron and/or simvastatin to treatment as usual.

**Trail registration:**

ClinicalTrails.gov NCT01602029

## Background

Although dopamine remains the primary neurotransmitter implicated in pathogenesis of schizophrenia, there is growing evidence to support inflammatory processes to be involved as well [1]. A number of studies have reported increased peripheral inflammatory cytokines (interleukin-1 (IL-1), IL-2, IL-6, and tumor necrosis factor alpha) [2] and elevated IL-1 in cerebrospinal fluid (CSF) of patients with schizophrenia [3]. In the brain, cytokines seem to be involved in regulating the action of several neurotransmitters, such as serotonin, noradrenaline, dopamine and glutamate. The interaction of cytokines with dopamine and glutamate seems to be especially relevant to the pathophysiology of schizophrenia [4].

Currently available treatments for schizophrenia are effective in treating positive symptoms, but have little or no impact on primary negative symptoms [5]. Negative symptoms and cognitive deficits are two partially-related features of schizophrenia which have a major negative impact on social function and objective quality of life [6]. Social dysfunction has major economic consequences in both the developed and developing world.

There is evidence that anti-inflammatory treatment may have beneficial effects in schizophrenia. In preliminary clinical trials for treatment of schizophrenia, Cox-2 inhibitors have shown favorable effects [1,7]. In our recent randomized double-blind placebo-controlled clinical trial in Pakistan and Brazil, the addition of minocycline (an antibiotic and anti-inflammatory drug) for one year to treatment as usual (TAU) reduced negative symptoms and improved some cognitive measures [8].

Statins are primarily HMG-CoA reductase inhibitors but are also anti-inflammatory agents. Similar to the Cox-2 inhibitors, they have the potential to improve these symptoms of schizophrenia [9]. The statins are also known to decrease C-reactive protein (CRP), which has been shown in an SMRI-funded study to be elevated in schizophrenia. Fan *et al.*[10] demonstrated in patients with schizophrenia higher levels of CRP (>0.50 mg/dl) to be associated with marked negative symptoms and higher total positive and negative syndrome scale (PANSS) scores.

Ondansetron, a selective 5-hydroxytryptamine-3 antagonist, is used quite commonly as an antiemetic in cancer patients [11]. There are several small trials suggesting that ondansetron, as an adjunct to antipsychotics, is effective in improving negative symptoms and memory in patients suffering from schizophrenia [12-14]. There is also evidence that 5HT3 antagonists have some anti-inflammatory effects through inhibition of TNF-alpha/interlukin-1beta secretions in human monocytes [15].

### Preliminary study

We conducted a small, placebo-controlled, rater-blind 12-week feasibility study adding ondansetron or simvastatin to TAU in 36 patients with chronic schizophrenia (ClinicalTrails.gov NCT00929955). Statins and ondansetron are well tolerated and their side effect profiles well understood. The aim of this study was to examine the tolerability of both compounds and allow estimation of effect sizes to power a larger trial. Both treatments were well tolerated and no serious side effects were reported.

### Aims

We propose to test the prediction that addition of ondansetron and/or simvastatin to TAU for patients with schizophrenia will result in the following outcomes:

•Primary: improvement in negative symptoms

•Secondary:

improvement in positive or other symptoms

improvement in social functioning

improvement in cognitive functions

possible additive effects of ondansetron and simvastatin added to TAU

## Methods/design

### Overview

This is a two center, six-month, double-blind placebo-controlled, factorial design study of ondansetron and/or simvastatin added to treatment as usual for patients suffering from schizophrenia, schizoaffective disorder, psychosis not otherwise specified or schizophreniform disorder. This will be a 2 × 2 factorial design, with 54 patients in each cell, giving a total of 216 patients over three years (Table 1).

**Table 1:** 2 × 2 Factorial design

	**Placebo**	**Simvastatin**	**Total**
Placebo	54	54	108
Ondansetron	54	54	108
Total	108	108	

The advantage of a factorial design is the effective sample size for each drug is 108 for each treatment factor. However, in addition it allows a test of scientifically and clinically interesting possibility that the two drugs have additive or even multiplicative benefits. A possible minor disadvantage is that all participants take two types of pills.

The study will be conducted in Karachi, Pakistan and San Paulo, Brazil. The patients will be recruited both from inpatient and outpatient settings. The patients will be identified and first approached by the treating psychiatrists and the multi-disciplinary teams (MDT). The patients will continue to take treatment as usual during the trial. While the study assessments will be done by the research assistants (RAs), the responsible psychiatrist will remain in charge of the overall treatment. Any changes to dose or types of medications will be recorded on monthly reviews.

After consenting to take part in the study, the clinical symptoms, cognitive, neurological and general level of function of the participants will be assessed. When these measures are completed, the patient will be randomized to one of the four treatment arms. During the course of the study the patient will also have additional support by doctors and RA from the research team and continued support from their mental health care team. If necessary, members of the research team will be available 24 hours a day, 7 days a week.

### Allocation

In this study patients will be allocated to treatment group according to a randomized permuted blocks algorithm, after stratification by center. Allocation will be determined in Manchester by pseudo-random number generation by the trial statistician. The results will be conveyed to the trial pharmacist so that medication can be given to participants without anyone else on the team being aware of the allocation (that is, double blind).

### Power calculation and statistical analysis

Our previous experience with trials in this area [8] and the pilot lead us to expect 20% dropout across all cells. In that case, we will have outcomes on 43 participants within each cell of the design. Assume, for the sake of illustration, that the mean PANSS negative score in the cell without either drug is 20 points after six months. Also assume that the single-drug-only cells (B and C) both have a mean PANSS negative score of 18 points. Finally, after treatment with both drugs (D), mean outcome score is 17 points (that is, a departure from additivity). The within-cell standard deviation is taken to be about five units. Using a two-way analysis of variance with a significance level of 0.05, the trial will have about 45% power to detect departures from additivity of this size. It will have about 80% power to detect differences between the cells with and without ondansetron in the cells without simvastatin (that is, A versus C), and, similarly, the differences with and without simvastatin in the two cells without ondansetron (A versus B). The following is based on the assumption that the two treatment effects are, indeed, additive (no interaction). When testing the effects of ondansetron, for example, the contrast will compare the mean of the 86 participants allocated to receive this drug with the mean of the 86 who were not (C and D versus A and C), and the trial will have over 90% power to detect the overall effects based on this contrast (Table 2).

**Table 2:** Statistical analysis plan

	**Placebo**	**Simvastatin**	**Total**
Placebo	A	B	(A + B)/2
Ondansetron	C	D	(C + D)/2
Total	(A + C)/2	(B + D)/2	

All analyses will be based, as far as possible, on the intention-to-treat principle (with weighting adjustments to allow for differential loss to follow-up – see below). Analysis of the three- and six-month quantitative outcomes will be based on an analysis of covariance (ANCOVA), using relevant baseline score and center membership as covariates. In line with the factorial design, we will initially evaluate the interaction between ondansetron and simvastatin. If this (as expected) is not statistically-significant, we will proceed to estimate the main effects of each of these two drugs separately (that is, in terms of the 2 × 2 design in the table above, by comparison of row means for ondansetron and column means for simvastatin, respectively). If the interaction was found to be statistically-significant the analysis would involve the estimation of all of the parameters of the saturated model. Patterns of missing outcome data will be examined as a function of recorded baseline characteristics and patterns of compliance with the treatment protocol. Adjustments will be made to allow for biases arising from patterns of missing outcome data linked to protocol violations using inverse-probability weighting.

### Inclusion criteria

1. Patients aged 18 to 65 years

2. Patients will be recruited both from inpatients and outpatients.

3. Diagnostic and Statistical Manual-IV (DSM-IV TR) diagnosis of schizophrenia, schizoaffective disorder, psychosis not otherwise specified or schizophreniform disorder

4. Competent and willing to give informed consent

5. Stable on medication four weeks prior to baseline

6. No planned medication change

7. Able to take oral medication and likely to complete the required evaluations

8. Female participants of child bearing age must be willing to use adequate contraceptives for the duration of the study and be willing to have a pregnancy test pre-treatment and at 10 weekly intervals while on study medication,

9. Not planning to relocate in the next 12 months

### Exclusion criteria

1. Relevant International Statistical Classification of Diseases and Related Health Problems (ICD)-10 organic brain disease or neurological diagnoses (including ECG conduction abnormalities, neurological disorder or an active seizure)

2. Subjects who will meet the criteria for a DSM-IV TR diagnosis of alcohol or substance abuse (other than for nicotine) within the last month or the criteria for DSM-IV TR alcohol or substance dependence (other than for nicotine) within the last six months

3. Any change of psychotropic medications within the previous four weeks

4. Pregnant or lactating women and those of reproductive age without adequate contraception

### Interval of assessments

There will be a screening, a randomization and seven follow-up visits. Full clinical and neurocognitive assessments will be carried out at baseline (randomization), 14 weeks and at 26 weeks, while PANSS, pill count and side effects checklist will be carried out at every visit. The clinical interview and ratings will take approximately 50 minutes. The neuropsychological assessments will take about 60 to 80 minutes. The patients will primarily be seen at their treating team’s base. In special circumstances, if requested by the treating team or patient, the RAs can visit at home (Table 3).

**Table 3:** Schedule of assessments

**Assessment**	**Who**			**When**								
	**Pt.**	**RA**	**RMO**	**Scr.**	**Rand.**	**W 2**	**W 6**	**W10**	**W14**	**W18**	**W22**	**W26**
**Case note review**			x									x
**SCID**		x		x								
**Drug treatment history**		x		x	x	x	x	x	x	x	x	x
**Medical history**			x	x								
**Physical exam**		x		x								x
**Body weight and BMI**		x			x				x			x
**BP and HR**		x		x	x	x	x	x	x	x	x	x
**Lab screen**			x	x					x			x
**Pregnancy screen (urine)**		x			x			x	x			x
**Inclusion criteria**		x		x								
**Exclusion criteria**		x		x								
**Withdrawal criteria**		x			x	x	x	x	x	x	x	
**Consent**	x	x		x	x							
**PANNS**		x			x	x	x	x	x	x	x	x
**CGI**		x			x	x	x	x	x	x	x	x
**GAF**		x			x				x			x
**QOL**		x			x				x			x
**Social function Scale**		x			x				x			x
**Cognitive assessments**		x			x				x			x
**Antipsychotic side effects**		x			x	x	x	x	x	x	x	x
**Side effects simvastatin/ondansetron**	x	x			x	x	x	x	x	x	x	x
**Compliance monitoring**		x				x	x	x	x	x	x	x

(Pt. Patient; RA, Research Assisstant; RMO, Reseident Medical Officer; W2, Week2; W6, Week 6; W10, Week10; W14, Week14; W18, Week18; W22, Week22; W26, Week26).

### RA training and interrater reliability

Research assistants in Karachi were trained in Structured Clinical Interview for DSM-IV (SCID), clinical and neuropsychological assessments at the University of Manchester for a previous SMRI funded study. Interrater reliability sessions will be conducted by local PIs, using training videos.

### Treatments

#### Treatment as usual

The patients can either be on first or second generation antipsychotic medications, as deemed suitable by the responsible psychiatrist. From our experience with previous study across the two centers in 144 patients, the distribution was approximately 50:50 between the two generations of antipsychotics.

#### Study drug dosage

Simvastatin will be started at 20 mg OD, this will be increased to 40 mg after four weeks. The dose range for hypercholesterolemia is from 10 mg to 80 mg once daily. We are not concerned with this drug’s performance in this indication; therefore, we consider 40 mg taken as one daily dose appropriate for the purposes of this research study. Statins have been used commonly with psychotropics for many years.

Ondansetron will be administered in an 8 mg dose. British National Formulary (BNF) specifies an 8 mg once daily dose. Studies by Akhondzadeh *et al.*[12] and Levkovitz *et al.*[13] have used 8 mg of ondansetron added to TAU with good tolerability in patients suffering from schizophrenia. Ondansetron is well tolerated, has no significant drug interaction reported, is broken down by cytochrome P450 and has little effect on metabolism of other drugs. It is often combined with psychotropic medication. There is no known indication of unexpected clinically significant interactions.

### Outcome variables

#### Primary clinical outcome variable

Negative symptom severity is defined by the negative syndrome subscale score on the Positive and Negative Syndrome Scale, which is the gold standard for comprehensively rating symptoms of schizophrenia [16].

#### Secondary clinical outcome variables

1. Full PANSS and positive syndrome subscale score [16]

2. Clinical Global Impression

3. Functional outcome:

•Global Assessment of Function (GAF) from DSM-IV [17]

•Social Functioning Scale (SFS) self-rating in seven domains [18]

•Quality of Life Scale (QLS) for treatment effects related to deficit or negative symptoms [19]

### Secondary cognitive outcome measures

We will use pencil and paper tests and the Cambridge Neuropsychological Test Automated Battery (CANTAB); measuring all seven domains recommended by MATRICS (NIMH initiative). These domains include speed processing, attention/vigilance, working memory (nonverbal and verbal), verbal learning, visual learning, reasoning and problem solving, and social cognitions. We will use the following tests:

1. IQ: using WAIS Block design

2. Stroop Task, for divided attention and processing speed

3. Coughlan Learning Task (Verbal and Visual)

4. Verbal Fluency (words and categories)

5. CANTAB

•Intra-Extra Dimensional Set Shifting (IED), which tests rule acquisition and attentional set shifting

•Spatial Working Memory (SWM), which tests working memory and strategic planning.

•Paired Associates Learning (PAL), which assesses episodic memory and learning

#### Side effects

Self-reported antipsychotic side-effects: Antipsychotic Non-neurological Side-Effects Rating Scale [20].

### Safety assessments

#### Medical history

The primary purpose of screening is to check for inclusion and exclusion criteria. The RAs will arrange for a doctor to complete checklists for medical history, review of systems, a physical exam and arrange a blood draw for hematology and differential white cell count, and for clinical chemistry to confirm normal renal and liver function. These assessments will be repeated after six months in the trial or at the time of withdrawal from the study.

#### Physical assessments

Physical assessments will include ECG, Blood pressure, pulse, height, waist, weight and body mass index (BMI).

#### Blood investigations

Blood investigations will include full blood count, erythrocyte sedimentation rate, liver function tests, lipid profile, urea, electrolytes and C-reactive protein.

#### Adverse events

An adverse event (AE) is the development of an undesirable medical condition or the deterioration of a pre-existing medical condition following or during exposure to a pharmaceutical product, whether or not considered causally related to the product. An undesirable medical condition can be symptoms (for example, nausea, chest pain), signs (for example, tachycardia, enlarged liver) or the abnormal results of an investigation (for example, laboratory findings, electrocardiogram). In clinical studies, an AE can include an undesirable medical condition occurring at any time, including run-in or washout periods, even if no study treatment has been administered.

The open question “Have you had any health problems since you were last asked?” will be used to evoke spontaneous reports of side effects. A self-completed checklist of adverse events derived from those specified in the data sheets for ondansetron and simvastatin will also be administered. Spontaneous and self-rated side-effects will be evaluated for intensity and seriousness.

### Study procedures

#### Recruitment

The psychiatry consultants responsible for patient care will be approached and asked if they will allow their patients to take part in this research study.

In the first instance, the research clinician will approach the clinical teams to inform them about the research study, especially with regard to inclusion and exclusion criteria. The research clinician will establish good working relationships with the individual clinical teams. They will be in regular contact either by phone or by visits to determine, in collaboration with the clinical team, if patients are suitable to take part in the research study. If patients meet the entry criteria, are clinically stable, and the consultant psychiatrist and the clinical team agree the patient could be a possible participant, the consultant will introduce the study to the patient. With the patient’s agreement, the research clinician will visit the patient to explain the research study verbally and provide them with the patient information sheet. After the patient has had time to read and understand the patient information sheet (at least 24 h) and is willing to take part, a meeting (visit one) will be set up with the patient in order to obtain consent for the research and also consent for the research team to have access to their medical notes.

#### Screening visit

Confirmation of patient suitability will be carried out at this point. This will take the form of checking the Inclusion/Exclusion criteria, confirmation of diagnosis, confirmation of consent and pregnancy testing, if appropriate. The RAs will arrange for a doctor to complete checklists for medical history, physical examination and hematological investigations.

#### Randomization visit

The baseline clinical and neuropsychological measures will be completed and the patient will be randomized to one of the four study arms, allocated a unique identification number and will commence study drug treatment.

The patient will be given a study information card explaining that they are in a clinical study and are taking simvastatin or ondansetron, the telephone numbers of the senior research clinicians, the clinical trial office and the name of the local PIs.

In accordance with (International Conference for Harmonisation) Good Clinical Practice ((ICH)GCP) copies of all the above will be placed in the patient’s medical notes along with contact names and telephone numbers.

##### Follow-up visits

The first follow-up visit will be after two weeks of the randomization to check for safety and tolerability of the treatment initiated. There will be visits at week 6, 10, 14, 18 and 22. On all these visits the patients will collect their trial treatment from their treatment centers. The study drugs at the two sites will be dispensed from the local pharmacy departments. The research assistants will collect and deliver these to the patient at a previously agreed upon location. This will give the opportunity for the research team to closely monitor the patient’s physical and mental health, side effects and compliance (by pill count).

Female patients with childbearing capacity taking part in the clinical trial will have a pregnancy test on a regular basis. If they should become pregnant during the course of the study, they will be withdrawn from the study.

During the course of the study the patient’s individual consultant and mental health care team will be responsible for their overall care. If any concerns do occur with regard to the research study, the research team can be contacted to discuss these concerns 24 h a day 7 days per week. The patient will have their study card with the telephone number of the research clinicians and clinical trial office if they have any concerns or questions.

One of the co-investigators and/or the study doctor will be available 24 h per day 7 days per week and will be contacted by the patient or carers or the research clinicians should this be necessary.

#### 26 week, final visit

At this visit all clinical, neuropsychological, physical and hematological assessments will be completed. Trial medication will cease.

At all visits the patient’s cumulative clinical drug treatment will be updated from the case notes.

#### Study coordination

The study will be coordinated by weekly meetings by local investigators. There will be two weekly tele- or video-conferences with the chief investigator. We are also able to use the access grid facility at the University of Manchester.

#### Patient safety

The patients’ day-to-day care is the responsibility of the consultant in charge of the patient. Any study related safety concerns are the responsibility of the local principal investigators (P.I.). The P.I.s and the co-investigators can be contacted at any time via the clinical trials office or/and through the senior research clinicians.

#### Patient withdrawal

Patients may be withdrawn from the treatment study for the following reasons:

1. At their own request.

2. At the discretion of the investigator.

3. If any patients suffers a serious adverse event, or moderate to severe adverse drug reaction.

4. Any patients meet the criteria for insufficient compliance. This is defined as either taking less than 75% of antipsychotic or trial medication between assessment points at baseline, 14 and 26 weeks; or missing trial medications or antipsychotic altogether for seven days or more at any period.

5. If a patient becomes pregnant.

They may withdraw from the study entirely (that is, follow-up) at their own request or at the investigator’s discretion.

### Ethical and regulatory standards

#### Local Research Ethics Committee (LREC)

Ethical approval covering all sites was obtained from the research and ethics committee of the Pakistan Institute of Learning and Living and was submitted to the funding body, Stanley Medical Research Institute.

#### Data and Safety Monitoring Board (DSMB)

The DSMB will be constituted according to NIH guidelines, for oversight and monitoring of the conduct of clinical trial. DSMB will meet every six months to ensure the safety of participants and the validity and integrity of the data.

#### Declaration of Helsinki

The research study will be performed in accordance with the guidelines in the Declaration of Helsinki (1974) as revised in Tokyo (1975), Venice (1983), Hong Kong (1989), South Africa (1996) and Scotland (2000).

All members of the research team will comply with International Conference on Harmonisation/Good Clinical Practice (ICH/GCP) Guidelines (1996) which are consistent with principles which have their origin in the Declaration of Helsinki.

#### Team expertise

Six of the investigators have extensive experience in treatment and management of schizophrenia (Chaudhry, Husain, Hallak, Drake, Kazmi, Rahman and Deakin). Kazmi and Rahman as local investigators will be important components of success in recruitment. All investigators have been closely involved in setting up the methodology and software for gathering clinical and research data in patients with psychosis. Deakin, Chaudhry, Husain, Stirling and Drake will oversee the training of RAs in recruiting patients and in diagnostic and symptom ratings. All of the investigators were involved in feasibility study for this trial. Deakin, Drake and Chaudhry are experts in psychopharmacology. Chaudhry, Husain, Drake and Hallak have been PIs in a number of multicentre trials funded by MRC and SMRI in schizophrenia and have much experience of large-scale treatment studies in psychosis. Drake, Stirling, Chaudhry, Hallak and Husain have published longitudinal studies on negative symptoms, cognition and quality of life. Husain, Chaudhry, Hallak, Kazmi, Rahman, Stirling and Deakin ran the SMRI trial of minocycline. Stirling is a reader in psychology and has particular expertise in neuropsychological testing in schizophrenia. Dunn is internationally known for his work on clinical trial statistical methodology and has published extensively. He has led the statistical trial design and analysis in many multicenter clinical trials, including in the treatment of schizophrenia. He has formal links with the Christie Hospital Clinical Trials Unit.

## Discussion

Schizophrenia is characterized by disorder of thought, perception, volition, socialization and psychomotor behavior. During the clinical course of the illness, deterioration is manifested with increased severity and persistence of negative and positive symptoms. Standard pharmacological treatments have little impact on negative symptoms leading to diminished social and functional outcome. Anti-inflammatory treatments have been shown to have some beneficial effects in schizophrenia. Both simvastatin and ondansetron provide some evidence of a reduction in symptoms compared to treatment as usual.

The preliminary feasibility study adding ondansetron or simvastatin to TAU in patients with chronic schizophrenia (ClinicalTrails.gov NCT00929955) showed both treatments were well tolerated and no serious side effects were reported. Findings are from this randomized controlled trial of simvastatin and ondansetron added to TAU may contribute to evidence in the management of negative symptoms and cognitive deficits in patients with schizophrenia.

## Trial status

The study is active and currently recruiting patients. We anticipate completing recruitment by the end of 2012 and final assessments completed by June 2013 (Table 4).

**Table 4:** Timeline

**Milestones (months)**	**–3**	**1**	**3**	**4**	**5**	**6**	**10**	**16**	**24**	**28**	**30**	**32**	**34**	**36**
Setting up/ethical approval	X	X	X											
Staff recruitment/training		X	X	X										
Patient recruitment/11 per month				X	X	X	X	X	X					
Intervention				X	X	X	X	X	X	X	X			
Data entry				X	X	X	X	X	X	X	X	X		
Write up and analysis											X	X	X	X

## Abbreviations

AE: Adverse Events; ANCOVA: Analysis of Covariance; BMI: Body Mass Index; BNF: British National Formulary; BP: Blood Pressure; CANTAB: Cambridge Neuropsychological Test Automated Battery; CGI: Clinical Global Impression; CRP: C-reactive protein; CSF: cerebrospinal fluid; DSMB: Data and Safety Monitoring Board; DSM-IV TR: Diagnostic and Statistical Manual-IV; ECG: Electro Cardio Gram; GAF: Global Assessment of Functioing; GCP: Good Clinical Practice; ICD-10: International Statiscal Classification of Diseases and Related Health Problems; ICH: International Conference for Harmonisation; IED: Intra-extra Dimensional Set Shift; HR: Heart Rate; LREC: Local Research Ethics Committee; MATRICS: Measurement and Treatment Research to Improve Cognition in Schizophrenia; MDT: Multi-Disciplinary Teams; MRC: Medical Research Council; NIMH: National Institute of Mental Health; OD: Once Daily; PANSS: Postive and Negative Syndrome Scale; Pt.: Patient; PI: Prinicipal Investigator; QLS: Quaility of Life Scale; QOL: Quality of Life; RA: Research Assisstants; RMO: Resident Medical officer; SFS: Socal Functioing Scale; SCID: Structured Clinical Interview for DSM-IV; SMRI: Stanley Medical Research Institute; SWM: Spatial Working memory; PAL: Paired Associates Learning; TAU: Treatment As Usual; WAIS: Wechsler Adult Intelligence Scale; W2: Week2; W6: Week 6; W10: Week10; W14: Week14; W18: Week18; W22: Week22; W26: Week26.

## Competing interests

IBC, BD, JH and NH have given lectures and advice to Lilly, Bristol Myers Squibb, Lundbeck, Astra Zeneca and Janssen pharmaceuticals for which they or their employing institution have been reimbursed. RR and MMH and AK have received educational grants and support for academic meetings from Pfizer, Roche, Novartis and Nabiqasim. None of the companies have a financial interest in this research. GD, JS, RD, TK and NM have no biomedical financial interests or potential conflicts of interest.

## Authors’ contributions

BD, IBC, NH, RD, JS and GD conceived the idea for the study and with JH, MMH, RR and AK contributed to the design of the study. JH, MMH, RR and AK are contributing to recruitment of the patients and, with TK and NH, are doing the assessments. IBC, NH, JH and RR are involved with training and inter-rater reliability. All authors, except JH and TK, were involved in the pilot study. All authors read and approved the final manuscript.
